# Stiff-person syndrome coexisting with critical illness polyneuropathy: A case report

**DOI:** 10.1097/MD.0000000000023607

**Published:** 2020-12-11

**Authors:** Qiong Cai, Chao Wu, Wenxiao Xu, Yinxing Liang, Songjie Liao

**Affiliations:** Department of Neurology, The First Affiliated Hospital, Sun Yat-sen University, Guangdong Provincial Key Laboratory of Diagnosis and Treatment of Major Neurological Diseases, National Key Clinical Department and Key Discipline of Neurology, Guangzhou, China.

**Keywords:** acute respiratory, critical illness polyneuropathy, electrophysiology, failure, muscle stiffness, stiff-person syndrome

## Abstract

**Rationale:**

Stiff-person syndrome (SPS) is an uncommon neurological disorder with autoimmune features. Here, we report a 60-year-old man with SPS associated with critical illness polyneuropathy (CIP). CIP was diagnosed during an episode of acute respiratory failure secondary to muscular rigidity and spasms, which has rarely been reported in this condition. The overlapping of CIP and SPS complicated the case.

**Patient concerns:**

A 60-year-old man presented with gradual onset of cramps, stiffness, and rigidity in his lower limbs 1 year before admission, which eventually led to inability to stand and walk. The persistent nature of his symptoms progressed to frequent acute episodes of dyspnea and he was admitted to intensive care unit (ICU).

**Diagnosis:**

SPS had been diagnosed after 2 tests of electromyography (EMG) and the detection of an elevated anti-GAD65 antibody titer. During the first EMG, low or absent compound muscle action potentials (CMAP), and sensory nerve action potentials (SNAP) were shown. Therefore, the diagnosis of SPS coexisting with CIP was made.

**Interventions:**

Symptomatic treatment was initiated with oral clonazepam (0.5 mg Bid) and baclofen (5 mg Bid). Intravenous immunoglobulin (IVIG) (0.4 g/kg/d) was administered for the patient for 5 days after admission. We observed a significant clinical improvement during the administration period, and the patient became ambulatory.

**Outcomes:**

On follow-up, the patient reported complete relief of his pain and rigidity.

**Lessons:**

We report this special case to address the varied clinical features of SPS. Electrophysiological testing is an important diagnostic approach. Accurate recognition of the disease ensures that the patients can be given appropriate treatment without delay.

## 1 Introduction

Stiff-person syndrome (SPS), an uncommon and disabling disorder autoimmune features, is characterized by progressive severe muscle stiffness and episodic spasms involving the spine and lower extremities. It initially affects the axial muscles and spreads to limb muscles in most cases, leading to chronic pain, spasms, postural deformities, and impaired motility. Emotional stress and sensory stimulation may elicit spasms of the legs and trunk or exacerbate clinical manifestations of the disease.^[1,2]^ Although the exact pathogenesis is unclear, between 60% and 80% of patients with SPS have serum antibodies to glutamic acid decarboxylase (GAD), the rate-limiting enzyme for the synthesis of gammaaminobutyric acid (GABA), an important inhibitory neurotransmitter of the brain and spinal cord. Up to 20% have the paraneoplastic variant where patients have associated neoplasms. The remaining 10% of patients are cryptogenic SPS.^[3]^

Critical illness polyneuropathy (CIP) is a neuromuscular disorder affecting 30% to 70% of critically ill patients. It has been reported that 26% to 65% of patients who require mechanical ventilation progressed to flaccid quadriparesis; the longer the patients are ventilated, the higher incidence of muscle flaccid weakness.^[4]^ And other studies demonstrated that mechanical ventilation is a risk factor for the emergence of CIP. Clinical features are generalized or distal weakness, flaccidity, and distal sensory deficits. The electrodiagnostic findings of CIP are a severe motor and sensory polyneuropathy, primarily affecting the lower extremity. Low or absent amplitude of both motor and sensory nerves are common. Usually, the nerve conduction velocity is in the normal range.^[5]^ The incidence of CIP in critically ill patients make it imperative to recognize the neuromuscular etiologies and prevent the development of neuromuscular weakness. Here we present a unique case of SPS with CIP, where CIP was drastically improved upon diagnosis and management of SPS. However, when they coexist, the diagnosis is extremely challenging.

## 2 Case presentation

A 60-year-old man presented with gradual onset of cramps, stiffness, and rigidity in his lower limbs 1 year before admission, eventually leading to inability to stand and walk. He had episodic muscle stiffness or spasms of the lower extremities. Sound and touch stimulation would elicit spasms of the legs or exacerbate the symptoms. Seven months before admission, he was treated as having tetanus and received an injection of tetanus antitoxin at the local hospital. However, the persistent nature of his symptoms progressed to frequent acute episodes of dyspnea, associated with hypertonic stiffness of axial muscles, pneumonia, polypnea, hypoxemia, and hypoproteinemia. He was admitted to the intensive care unit (ICU) to receive mechanical ventilation, antibiotics, and sedation. He developed generalized weakness of the limbs, flaccidity, and hyporeflexia at 14 days after ICU admission. There was no sign of anisocoria or facial muscle paralysis. Brain MRI showed no abnormalities. He fulfilled the criteria used commonly for diagnosing CIP: critically ill; limb weakness is present; difficulty in weaning from mechanical ventilatory support with the exclusion of cardiac and pulmonary causes; electrophysiological evidence of axonal sensorimotor neuropathy; other causes of acute neuropathy should be excluded.^[6]^ Following intravenous immunoglobulin (IVIG) (25 g/d for 2 days) therapy, active rehabilitation and symptomatic treatment, his limb cramps and weakness had been improved. The patient was discharged with advice to continue active rehabilitation training. During the 2 months after discharge, stiffness gradually extended to facial muscles, leading to eating problems. Thus he was seen by the neurologist and was hospitalized.

The patient had a history of hypertension and upper gastrointestinal hemorrhage, a nail scratches on the head with bleeding. His family history and review of systems were noncontributory.

In the neurological examination, the movements of lower limbs were limited by stiffness in distal and proximal. Deep tendon reflexes were exaggerated, and no hyperlordosis or myoclonus was observed.

**Figure 1:**
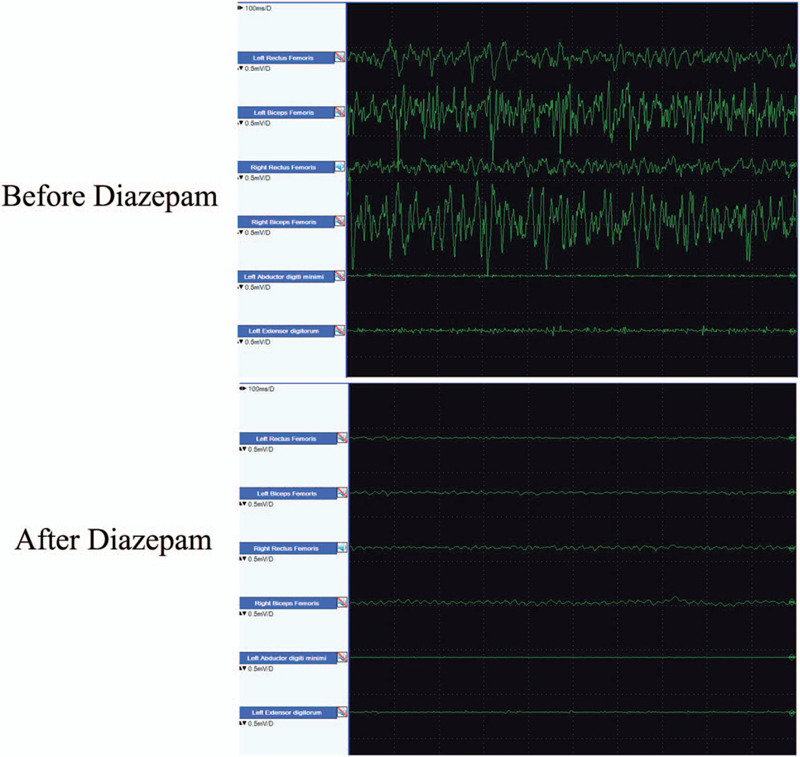
Presentive image of EMG. The upper panel shows simultaneous motor unit activity of bilateral rectus femoris and biceps femoris as agonistic and antagonistic muscles during relaxation. The muscles from the arm were recorded as control. The lower panel shows complete disappearance of muscle activity after intravenous injection of diazepam. EMG = electromyography.

Laboratory examination showed serum anti-GAD65 titer was high. Renal, hepatic functions, and immunologic factors were in normal range. The thyroid function tests showed normal levels of antithyroglobulin and thyroid-stimulating hormone receptor antibody. The patient's tumor markers and paraneoplastic antibodies were not elevated. MRI of the brain, electroencephalogram, and cerebrospinal fluid analysis were unremarkable. Nerve conduction study showed that the amplitudes of compound muscle action potentials (CMAP) and sensory nerve action potentials (SNAP) had markedly increased compared with the first nerve conduction study 6 months earlier (Tables 1 and 2). As there was no evidence of peripheral neuropathy, this further corroborated the proposed link between CIP and SPS in this patient. He underwent electromyography (EMG) which showed continuous normal motor unit action potentials (MUAPs) simultaneously occurred in both agonistic and antagonistic muscles of the lower limbs even in relaxation, and relieved by administration diazepam (10 mg) (Fig. 1). There were no other abnormal spontaneous potentials such as myokymia or myotonic discharge. Diagnosis of SPS was confirmed according to the criteria: a prodrome of episodic aching stiffness of axial muscles; progression to include stiffness of proximal limbs; painful spasms elicited by triggers; increased lumbar lordosis; normal sensation, motor function, and intellect; continuous motor-unit activity on electromyogram abolished by benzodiazepines; high-titer glutamic acid decarboxylase (GAD) antibodies.^[7]^

**Table 1:** Motor nerve conduction studies.

	Nerve	Segment	Latency, ms	Amplitude, mV	CV, m/s
The first examination	Ulnar, R	Wrist–elbow/ADM	2.81–8.31	^∗^0.75–0.77	52.7
	Median, R	Wrist–elbow/APB	3.66–7.94	^∗^1.21–1.0	51.4
	Tibial, L	Ankle–knee/AH	4.07–12.5	^∗^3.7–3.4	42.7
	Tibial, R	Ankle–knee/AH	4.23–13.2	^∗^3.4–3.0	36.8
	Peroneal, L	Ankle–knee/EDB	^∗^NE		
The second examination	Ulnar, R	Wrist–elbow/ADM	2.25–7.23	13.7–13.3	54.2
	Median, R	Wrist–elbow/APB	3.61–7.02	7.4–6.4	58.7
	Tibial, L	Ankle–knee/AH	4.13–11.9	6.5–6.2	43.1
	Tibial, R	Ankle–knee/AH	4.15–11.7	6.7–4.5	45.7
	Peroneal, L	Ankle–knee/EDB	^∗^NE		

The CMAPs in the ulnar, median, and tibial nerves exhibited a markedly increased amplitude compared with those in the first examination. ADM = abductor digiti minimi, AH = abductor hallucis, APB = abductor pollicis brevis, CV = conduction velocity, EDB = extensor digitorum brevis, NE = not evoked.

∗ Abnormal finding.

**Table 2:** Sensory nerve conduction studies.

	Nerve	Segment	DL, ms	Amplitude, μV	CV, m/s
The first examination	Ulnar, R	Digit V	3.48	8.8	40.2
	Median, R	Digit III	3.72	5.9	44.9
	Tibial, L	Great toe	5.62	0.83	36.5
	Peroneal, L	Great toe	6.09	0.69	30.3
The second examination	Ulnar, R	Digit V	3.48	10.4	43.1
	Median, R	Digit III	3.63	7.4	49.6
	Tibial, L	Great toe	4.82	1.08	41.2
	Peroneal, L	Great toe	5.74	0.77	34.2

The SNAPs in all examined nerves exhibited a slightly increased amplitude and velocity compared with the first examination. CV = conduction velocity; DL = distal latency.

Symptomatic treatment was initiated with oral clonazepam (0.5 mg Bid) and baclofen (5 mg Bid). IVIG (0.4 g/kg/5 d) was administered for the patient 4 days after admission. We observed a significant clinical improvement during the administration period, and the patient became ambulatory. Six months later in the follow-up, the patient reported complete relief of his pain and rigidity. And there was no numbness or weakness in his limbs. He remained on oral clonazepam and baclofen for continued symptomatic relief.

## 3 Discussion

The first description of SPS was introduced by Moersch and Woltman in 1956, as an uncommon condition that affects women more than men.^[7]^ Usually the rigidity and stiffness begin insidiously in axial muscle and over time progress to the proximal muscles. For our patient, rigidity and spasticity began in lower distal limbs, spreading proximally and causing walking difficulties that are rare in SPS. And he was initially misdiagnosed as tetanus in the local hospital. Furthermore, some SPS patients may exhibit autonomic symptoms and crises including polypnea, hyperhidrosis, and hyperthermia. Repeated muscle spasms and stiffness may be accompanied by life-threatening and unpredictable autonomic failure, which necessitate emergent measures.^[1]^ Sudden and unpredictable deaths have been described in 10% of SPS patients, and most of the cases due to acute respiratory failure and apnea.^[8]^ In addition, paroxysmal spasms of the diaphragm and intercostal muscles may also impaire respiratory function, requiring mechanical ventilation.^[2,9]^ It was reported that acute respiratory failure can be associated with some SPS patients.^[10,11]^ Our patient suffered from stiffness and spasms of the respiratory muscles which required mechanical ventilation and ICU admission for continuous monitoring until immunotherapy took effect. This was considered as the cause of CIP, since mechanical ventilation is a risk factor for the emergence of CIP. Approximately 25% to 33% of critically illness patients with mechanical ventilation for 4 to 7 days exhibit CIP if they are evaluated by clinical examination, and up to 58% of the patients if they are evaluated by electrophysiological testing.^[12]^ CIP is characterized by a combination of distal weakness, flaccidity, distal sensory deficits, and variable atrophy. The electrophysiologic findings are characterized by a low or absent amplitude of both motor and sensory nerves without features of demyelination. The abnormalities are generally worser in the legs. In some studies, CMAPs were more often affected than SNAPs.^[13,14]^ The incidence of CIP in critically ill patients make it imperative for early mobilization and initiation of physical therapy to prevent prolonged muscle weakness. In the present case, our male patient, who suffered from stiffness and cramps, developed the CIP, giving rise to this complex clinical scenario. Because the severe peripheral nerve damage obscured the symptoms of SPS and the first needle EMG study failed to recruit the MUAPs due to the severe muscle weakness, the patient was misdiagnosed as having peripheral neuropathy without recognition of SPS when he was in the ICU.

Other differential diagnoses need to be considered. This patient had presented with symmetrical flaccid weakness of the extremities after a relatively long duration of mechanical ventilation. Electrophysiological findings showed motor and sensory nerve axonal injury without conduction block or temporal dispersion. Acute motor axonal neuropathy (AMAN) may show some similar clinical profile, but presents with a pure motor syndrome and motor axonal impairment without sensory involvement, as well as cytoalbuminological dissociation.^[15]^ The clinical manifestations of SPS can also be similar to those of Isaacs syndrome. However, Isaacs syndrome presents different needle electromyography features consisting of myokymia and fasciculations, indicating increased nerve fiber excitability,^[16]^ while the findings of SPS exhibits continuous normal motor unit firing in both agonistic and antagonistic muscles during relaxation without abnormal spontaneous potentials, disappearing after intravenous injection of diazepam.

Benzodiazepines are thought to be an effective therapy to modulate the level of GABA. Our case responds dramatically with baclofen, clonazepam, and IVIG.

Rituximab, an anti-CD-20 monoclonal antibody that targets B lymphocytes, has been occasionally reported to contribute to clinical improvement of SPS.^[17]^

In conclusion, SPS is an uncommon neurological disorder with autoimmune features, CIP associated with SPS is even more uncommonly encountered. We have described this unique case in an attempt to stress that the clinical features of SPS are varied, and the coexisting situation complicates the diagnosis. Electrophysiological tests can be a very important contribution to the diagnostic approach in this condition. It is imperative that we raise the index of suspicion for SPS and improve the likelihood of its earlier diagnosis and treatment.

## Acknowledgments

We are grateful to the patient who made this study possible. We also would like to thank Doctor Yew-Long Lo of Singapore General Hospital for his kind advice.

## Author contributions

**Conceptualization:** Songjie Liao.

**Data curation:** Wenxiao Xu, Yinxing Liang.

**Funding acquisition:** Songjie Liao.

**Investigation:** Wenxiao Xu.

**Methodology:** Yinxing Liang, Qiong Cai, Chao Wu.

**Supervision:** Songjie Liao.

**Validation:** Yinxing Liang.

**Writing – original draft:** Qiong Cai, Chao Wu.

**Writing – review & editing:** Songjie Liao.
